# The association between vision impairment and multi-site pain in middle-aged and older adults in China: results from the China health and retirement longitudinal study

**DOI:** 10.3389/fpubh.2025.1579774

**Published:** 2025-06-16

**Authors:** Tianyi Luo, Cunzi Li, Lan Zhou, Yingrui Liu, Hongyan Sun, Ming Ming Yang

**Affiliations:** ^1^Department of Ophthalmology, The Second Clinical Medical College of Jinan University, Shenzhen People’s Hospital, Shenzhen, China; ^2^Department of Ophthalmology, Shenzhen People’s Hospital (The First Affiliated Hospital, Southern University of Science and Technology, The Second Clinical Medical College, Jinan University), Shenzhen, China; ^3^Post-Doctoral Scientific Research Station of Basic Medicine, Jinan University, Guangzhou, China

**Keywords:** vision impairment, multi-site pain, coexistence of pain, China health and retirement longitudinal study, Chinese middle-aged and older adults

## Abstract

**Background:**

Previous research on the association between vision impairment (VI) and multi-site pain has been sparse, and no studies have specifically examined this relationship in low- and middle-income countries (LMICs).

**Objective:**

This study aims to investigate the relationship between VI and the coexistence of pain in 15 different body sites and multi-site pain among middle-aged and older adults in China using nationally representative survey data.

**Methods:**

We conducted a cross-sectional study using data from the 2020 China Health and Retirement Longitudinal Study (CHARLS), which included 10,240 participants. We used the Mann–Whitney U test and chi-square test to compare the sociodemographic, economic, and health status characteristics of the participants. Two logistic regression models were constructed to analyze the relationship between VI and the coexistence of pain in different body sites and multi-site pain.

**Results:**

Participants with VI had a higher probability of experiencing pain across 15 body sites compared to those without VI. After adjusting for sociodemographic, economic, and health status factors, pain in eight different body sites was significantly associated with VI (*p <* 0.05). The most significant associations were observed for waist pain (*p* = 0.003), finger pain (*p* = 0.012), and knee pain (*p* = 0.009). Furthermore, VI was inversely associated with the coexistence of pain in two body sites (OR: 0.547; 95% CI: 0.354–0.810, *p <* 0.05) but positively associated with five or more body sites (OR: 1.550; 95% CI: 1.191–2.017, *p <* 0.05). Sensitivity analysis revealed that VI remained positively associated with the coexistence of five or more painful body sites after stratifying by age, gender, and place of residence (*p <* 0.05).

**Conclusion:**

Our study revealed that Chinese middle-aged and older individuals with VI tended to experience multi-site pain, exhibiting a negative correlation with two coexisting painful sites and a positive correlation with five or more. The association between VI with the coexistence of five or more painful body sites was not influenced by age, gender, or place of residence. These findings suggest that in LMICs, VI often occurs with multi-site pain, and patients with pain could benefit from ophthalmic care and vision rehabilitation. This has major implications for improving healthcare efficiency, service planning, and clinical practice. However, VI was assessed in this study through interviewer observation rather than clinical examinations, which may have introduced misclassification bias, and future studies should validate these associations using objective visual assessments.

## 1 Introduction

Vision impairment (VI) is a major global public health concern, especially among middle-aged and older adults (1). The prevalence of VI has risen significantly with global population aging. By 2020, approximately 1.1 billion people worldwide were affected, and this number is projected to reach 1.7 billion by 2050 (2). Additionally, the prevalence of VI is notably higher in low- and middle-income regions than the global average (3). VI not only severely impacts the quality of life of affected individuals but also imposes substantial socioeconomic burdens.

Pain is another prevalent condition associated with aging. Studies show that approximately 70% of middle-aged and older adults with pain report pain in multiple body sites, with over 20% reporting four or more sites (4). This widespread pain not only affects daily activities but also leads to decreased sleep quality and mental health problems. Specifically, 35% of chronic pain patients suffer from depression, impairing social functioning (5). Furthermore, widespread pain is often associated with various chronic diseases, increasing medical and social costs globally (6, 7). Therefore, when VI coexists with widespread pain, it significantly affects patients’ quality of life and social functioning. It is crucial to further clarify the relationship between VI and widespread pain. Understanding their association will help optimize clinical treatment strategies, reveal underlying pathological mechanisms, and promote multidisciplinary collaboration, driving more comprehensive health intervention measure development.

However, previous studies on the association between VI and multi-site pain have been limited, focusing primarily on the relationship between VI and pain in a single body site or tissue. Moreover, there is a significant gap between low- and middle-income countries (LMICs) and high-income countries in the diagnosis and treatment of VI and pain management due to differences in lifestyle, income and expenditure levels, environment, and socioeconomic factors. This gap further exacerbates healthcare resource pressures in LMICs. To date, no studies have explored the coexistence of VI and multi-site pain in LMICs. Therefore, this study aims to utilize nationally representative survey data to analyze the relationship between VI and the coexistence of pain in 15 different body sites and multi-site pain among middle-aged and older adults in China.

## 2 Methods

### 2.1 Study design and population

This cross-sectional study utilized data from the China Health and Retirement Longitudinal Study (CHARLS). CHARLS is a longitudinal survey representative of individuals aged 45 and above in Mainland China, collecting high-quality quantitative and qualitative data through face-to-face interviews and structured questionnaires using a multi-stage, stratified, and proportionate sampling method (8). The national baseline survey was conducted in 2011–2012, followed by four rounds of follow-up surveys in 2013, 2015, 2018, and 2020, and a life-course survey of middle-aged and older adults in China in 2014. To ensure representativeness, the CHARLS baseline survey covered 150 counties/districts, 450 villages/urban communities, and 17,708 individuals from 10,257 households across China, reflecting the overall situation of middle-aged and older adults in China. The CHARLS project protocol adhered to the Declaration of Helsinki and was approved by the Peking University Biomedical Ethics Review Committee (IRB00001052-11015). All participants provided written informed consent before completing the survey.

**Figure 1:**
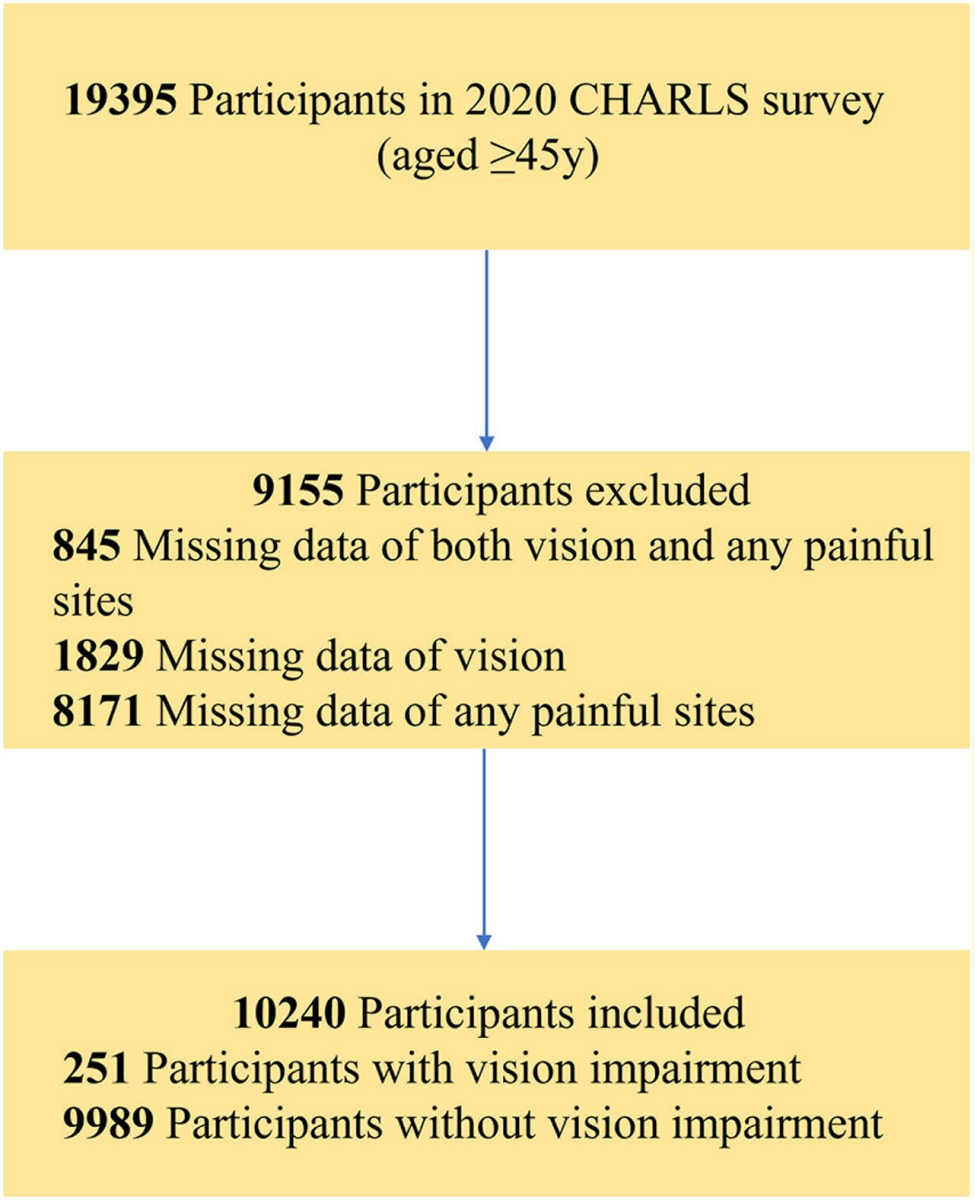
Flowchart depicting this study’s participant selection.

We used data from the 2020 CHARLS (with 19,395 participants, aged ≥ 45 years) to analyze the relationship between VI and multi-site pain. Participants lacking data on visual status and pain in any body site were excluded. Figure 1 illustrates the participant selection process. The final sample included 10,240 participants.

### 2.2 Definition of vision impairment

In the 2020 CHARLS data, VI was assessed by interviewers observing the visual status of respondents during the interview process. Interviewers were asked whether the respondent had poor eyesight during the interview: “0” for “no” and “1” for “the respondent had poor eyesight.” In this study, respondents with an answer of “1” to this question were classified as having VI.

Note that VI was assessed via interviewer observation without standardized clinical criteria (e.g., visual acuity testing, refraction, or ophthalmological diagnosis). Interviewers subjectively categorized participants as having ‘poor eyesight’ based on their interactions. This approach may introduce misclassification bias, as functional VI (e.g., uncorrected refractive errors) or mild/moderate impairment could be underreported. Additionally, the absence of standardized thresholds limits comparability with studies using objective measures. This is thus a major study limitation.

### 2.3 Definition of multi-site pain and coexistence of multi-site pain

This study used pain in various body sites (15 locations) to measure the coexistence of multi-site pain. Participants were asked to report the presence of pain in the following body sites: Head, Shoulder, Arm, Wrist, Fingers, Chest, Stomach, Back, Waist, Buttocks, Leg, Knees, Ankle, Toes, Neck; responses were coded as “0” for “no” and “1” for “yes.” Multi-site pain coexistence was defined as the presence of pain in two or more different body sites in a participant.

### 2.4 Covariates

The CHARLS questionnaire collected data on the sociodemographic and economic characteristics of participants, including age, gender (male or female), place of residence (city/town or village), marital status (married, never married, or divorced/widowed), smoking status (current smoker, former smoker, or never smoked), drinking status in the past year (drinking more than once a month, less than once a month, or never), health insurance status (insured or uninsured), household monthly expenditure (categorized into quartiles based on 25th, 50th, and 75th percentiles), and education level (primary school or below, or middle school or above). Data on participants’ health status included physical activity level (vigorous, moderate, light, or insufficient), self-reported health status (good, fair, or poor), and chronic disease status [ever told by a doctor they had hypertension, dyslipidemia (high or low lipid levels), diabetes or elevated blood glucose (including impaired glucose tolerance and elevated fasting blood glucose), malignant tumors (excluding mild skin cancer), chronic pulmonary diseases such as chronic bronchitis or emphysema, cor pulmonale (excluding tumors or cancer), liver diseases (excluding fatty liver, tumors, or cancer), heart diseases (such as myocardial infarction, coronary heart disease, angina pectoris, congestive heart failure, and other heart diseases), stroke, kidney diseases (excluding tumors or cancer), gastrointestinal diseases (excluding tumors or cancer), emotional and mental problems, memory-related diseases (Alzheimer’s disease, brain atrophy), Parkinson’s disease, arthritis or rheumatism, and asthma (non-pulmonary disease)]. The presence of any chronic disease was coded as “yes,” and the absence as “no.”

### 2.5 Statistical analysis

The Anderson-Darling test was utilized to evaluate the normality of continuous numerical variables. For non-normally distributed continuous variables (such as age in this study), data were presented as median (interquartile range). Categorical variables were presented as counts (percentages). The Mann–Whitney U test and chi-square test were utilized to compare sociodemographic, economic, and health status characteristics among participants. Two logistic regression models were constructed to estimate the association between VI and the coexistence of pain in different body sites and multi-site pain. Model 1 was adjusted for age and gender, while Model 2 further adjusted for place of residence, marital status, smoking status, drinking status, health insurance status, physical activity level, self-reported health status, chronic disease status, education level, and household monthly expenditure based on Model 1. To assess the robustness of the findings, we performed sensitivity analyses stratified by age group (45–60 years, ≥ 60 years), gender, and place of residence. All statistical analyses were performed using RStudio software (version 4.4.1). *p <* 0.05 was considered statistically significant.

We chose binary logistic regression to analyze the association between VI and the coexistence of pain in different body sites. While multinomial or ordered logistic regression could be considered for ordinal data, our study focused on specific patterns of pain coexistence (i.e., the presence of pain in two body sites or five or more body sites), rather than modeling the entire spectrum of pain site counts. Binary logistic regression provided a straightforward and interpretable approach to address our research questions while minimizing model complexity. Additionally, we performed sensitivity analyses to ensure the robustness of our findings.

## 3 Results

### 3.1 Participant characteristics

A total of 10,240 participants were included in this study, Table 1 presents their sociodemographic, economic, and selected health status characteristics. The median age of participants was 61 years (interquartile range, 14 years), and 38.9% were male. There were 251 participants with VI (2.5%). Overall, compared with participants without VI, those with VI were older, more likely to reside in rural areas, unmarried, never drinkers, without health insurance, reported poorer self-rated health, had lower levels of education, lower household monthly expenditure, and had a higher likelihood of having chronic diseases (all *p <* 0.05).

**Table 1:** Characteristics of participants with and without vision impairment.

Variable	Total	Vision impairment		*p*-value
		Yes	No	
No of participants	10,240	251	9,989	
Age, y	61 (14)	67 (15)	61 (14)	<0.001
Gender				0.528
Male	3,985 (38.9)	103 (41.0)	3,882 (38.9)	
Female	6,255 (61.1)	148 (59.0)	6,107 (61.1)	
Residence				0.001
City/Town	3,437 (33.6)	60 (23.9)	3,377 (33.8)	
Village	6,803 (66.4)	191 (76.1)	6,612 (66.2)	
Marital status				<0.001
Married	8,639 (84.4)	180 (71.7)	8,459 (84.7)	
Divorced/Widowed	1,550 (15.1)	66 (26.3)	1,484 (14.9)	
Never married	51 (0.5)	5 (2.0)	46 (0.5)	
Smoking status				0.534
Current	2,354 (23.0)	65 (25.9)	2,289 (22.9)	
Former	964 (9.4)	22 (8.8)	942 (9.4)	
Never	6,922 (67.6)	164 (65.3)	6,758 (67.7)	
Drinking status in the past year				0.005
Drink more than once a month	2,360 (23.1)	42 (16.7)	2,318 (23.2)	
Drink but less than once a month	1,017 (9.9)	17 (6.8)	1,000 (10.0)	
Never	6,863 (67.0)	192 (76.5)	6,671 (66.8)	
Health insurance				0.048
Insure	9,786 (95.6)	233 (92.8)	9,553 (95.6)	
Uninsured	454 (4.4)	18 (7.2)	436 (4.4)	
Physical activity				<0.001
Vigorous	3,986 (38.9)	76 (30.3)	3,910 (39.1)	
Moderate	3,268 (31.9)	66 (26.3)	3,202 (32.1)	
Light	2049 (20.0)	63 (25.1)	1986 (19.9)	
Insufficient	937 (9.2)	46 (18.3)	891 (8.9)	
Self-health report				<0.001
Good	1,378 (13.5)	19 (7.6)	1,359 (13.6)	
Fair	5,269 (51.5)	95 (37.9)	5,174 (51.8)	
Poor	3,593 (35.1)	137 (54.6)	3,456 (34.6)	
Chronic disease				0.042
Yes	4,437 (43.3)	125 (49.8)	4,312 (43.2)	
No	5,803 (56.7)	126 (50.2)	5,677 (56.8)	
Education level				<0.001
Primary school or below	7,324 (71.5)	209 (83.3)	7,115 (71.2)	
Middle school or above	2,916 (28.5)	42 (16.7)	2,874 (28.8)	
Monthly household expenditure				<0.001
Group 1	2,619 (25.6)	92 (36.7)	2,527 (25.3)	
Group 2	2,524 (24.7)	66 (26.3)	2,458 (24.6)	
Group 3	3,169 (31.0)	56 (22.3)	3,113 (31.2)	
Group 4	1928 (18.8)	37 (14.7)	1891 (18.9)	

Age data are presented as medians (interquartile ranges); categorical data are presented as number (%).

### 3.2 Prevalence of multi-site pain and coexistence of multi-site pain

**Figure 2:**
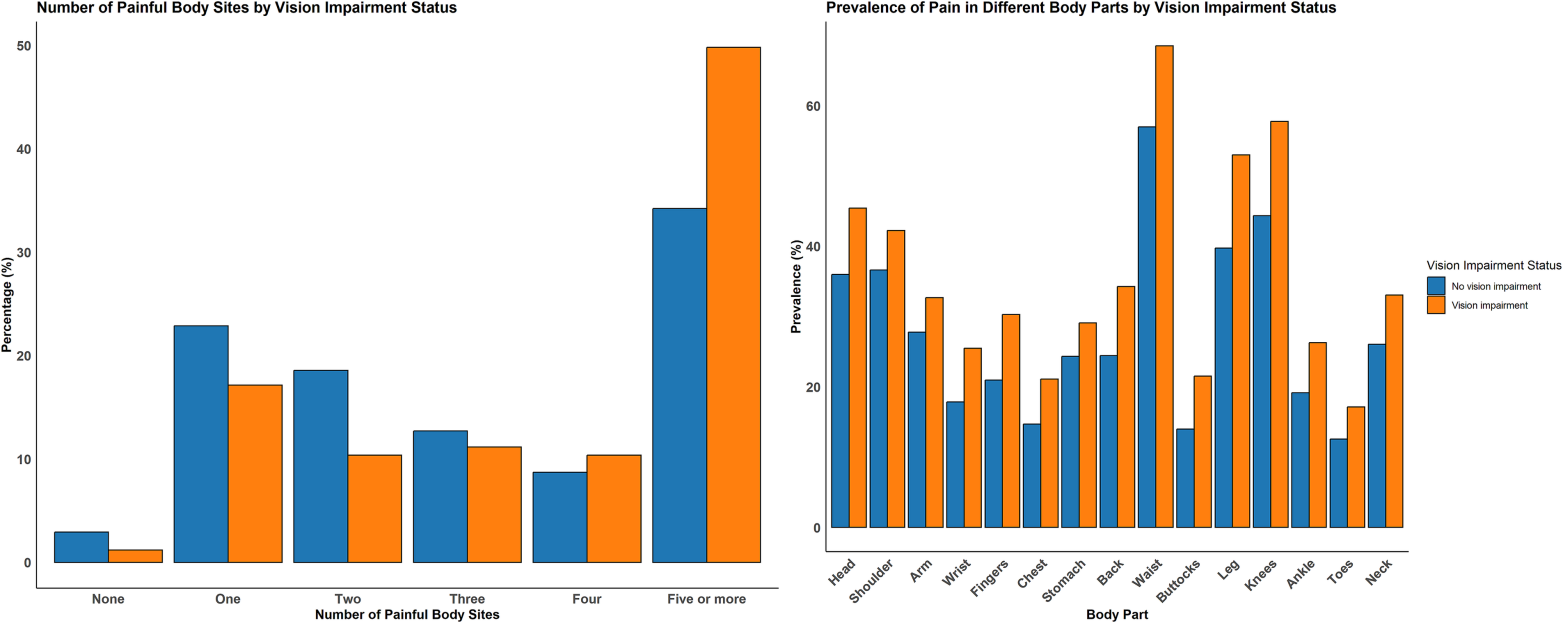
Prevalence of multi-site pain and coexistence of multi-site pain with and without visual impairment.

Figure 2 and Table 2 show the prevalence of multi-site pain and its coexistence with VI. Participants with VI had higher probabilities of pain in 15 different body sites than those without VI (all *p <* 0.001). Waist pain was most prevalent (68.5%) and toe pain was least prevalent (17.1%) among participants with VI. The prevalence of having four or more painful body sites was higher among participants with VI versus those without (*p <* 0.001). Among participants with VI, 49.8% had five or more painful body sites, compared to 34.2% among those without VI.

**Table 2:** Prevalence of multi-site pain and coexistence of multi-site pain among participants with and without visual impairment.

Variable / Pain location	Total	Vision impairment		*p*-value
		Yes	No	
Number of pain occurrences in different body parts				<0.001
None	296 (2.9)	3 (1.2)	293 (2.9)	
One	2,327 (22.7)	43 (17.1)	2,284 (22.9)	
Two	1881 (18.4)	26 (10.4)	1855 (18.6)	
Three	1,297 (12.7)	28 (11.2)	1,269 (12.7)	
Four	896 (8.8)	26 (10.7)	870 (8.7)	
Five or more	3,543 (34.6)	125 (49.8)	3,418 (34.2)	
Pain in different body parts				
Head	3,708 (36.2)	114 (45.4)	3,594 (36.0)	<0.001
Shoulder	3,765 (36.8)	106 (42.2)	3,659 (36.7)	<0.001
Arm	2,856 (27.9)	82 (32.7)	2,774 (27.8)	<0.001
Wrist	1845 (18.0)	64 (25.5)	1781 (17.8)	<0.001
Fingers	2,169 (21.2)	76 (30.3)	2093 (21.0)	<0.001
Chest	1,522 (14.9)	53 (21.1)	1,469 (14.7)	<0.001
Stomach	2,503 (24.4)	73 (29.1)	2,430 (24.3)	<0.001
Back	2,528 (24.7)	86 (34.3)	2,442 (24.4)	<0.001
Waist	5,864 (57.3)	172 (68.5)	5,692 (57.0)	<0.001
Buttocks	1,452 (14.2)	54 (21.5)	1,398 (14.0)	<0.001
Leg	4,103 (40.1)	133 (53.0)	3,970 (39.7)	<0.001
Knees	4,574 (44.7)	145 (57.8)	4,429 (44.3)	<0.001
Ankle	1978 (19.3)	66 (26.3)	1912 (19.1)	<0.001
Toes	1,297 (12.7)	43 (17.1)	1,254 (12.6)	<0.001
Neck	2,686 (26.2)	83 (33.1)	2,603 (26.1)	<0.001

Data are represented as the number (%).

### 3.3 Association between vision impairment and multi-site pain and coexistence of multi-site pain

Table 3 shows the association between VI and multi-site pain, as well as the coexistence of multi-site pain. After adjusting for age and gender, VI was inversely associated with the coexistence of two painful body sites (OR: 0.528; 95% CI: 0.342–0.782, *p <* 0.05) but positively associated with five or more painful body sites (OR: 1.802; 95% CI: 1.395–2.327, *p <* 0.001). No significant associations were observed for three (*p* = 0.511) or four (*p* = 0.456) painful body sites. VI was significantly associated with headache, shoulder pain, wrist pain, finger pain, chest pain, back pain, waist pain, buttock pain, leg pain, knee pain, and neck pain (all *p <* 0.05), but not arm pain (*p* = 0.106), stomach pain (*p* = 0.087), ankle pain (*p* = 0.051), or toe pain (*p* = 0.458). After further adjusting for other confounding factors, VI remained inversely associated with the coexisting pain in two body sites (OR: 0.547; 95% CI: 0.354–0.810, *p <* 0.05) while maintaining positive association with five or more sites (OR: 1.550; 95% CI: 1.191–2.017, *p <* 0.05). The odds of headache (OR: 1.321; 95% CI: 1.016–1.716), wrist pain (OR: 1.377; 95% CI: 1.016–1.843), finger pain (OR: 1.439; 95% CI: 1.079–1.904), back pain (OR: 1.330; 95% CI: 1.007–1.745), waist pain (OR: 1.518; 95% CI: 1.157–2.008), leg pain (OR: 1.316; 95% CI: 1.015–1.709), knee pain (OR: 1.419; 95% CI: 1.094–1.844), and neck pain (OR: 1.353; 95% CI: 1.023–1.778) were significantly higher among individuals with VI, with the most significant associations observed for waist pain (*p* = 0.003), finger pain (*p* = 0.012), and knee pain (*p* = 0.009). However, VI was not significantly associated with shoulder pain, arm pain, chest pain, stomach pain, buttock pain, ankle pain, or toe pain. Based on sensitivity analyses stratified by age group, gender, and place of residence, the association between VI and the coexistence of five or more painful body sites remained significant (*p <* 0.05) (Table 4).

**Table 3:** Association between visual impairment and multi-site pain and coexistence of multi-site pain.

Variable / Pain location	Model 1^*^		Model 2^**^	
	OR (95% CI)	*p*-value	OR (95% CI)	*p*-value
Number of pain occurrences in different body parts				
Two	0.528 (0.342–0.782)	<0.05	0.547 (0.354–0.810)	<0.05
Three	0.874 (0.574–1.281)	0.511	0.930 (0.610–1.366)	0.724
Four	1.172 (0.755–1.743)	0.456	1.177 (0.758–1.753)	0.445
Five or more	1.802 (1.395–2.327)	<0.001	1.550 (1.191–2.017)	<0.05
Pain in different body parts				
Head	1.511 (1.167–1.953)	<0.05	1.321 (1.016–1.716)	<0.05
Shoulder	1.306 (1.008–1.687)	<0.05	1.201 (0.923–1.557)	0.170
Arm	1.250 (0.950–1.633)	0.106	1.114 (0.843–1.461)	0.442
Wrist	1.576 (1.169–2.099)	<0.05	1.377 (1.016–1.843)	<0.05
Fingers	1.622 (1.221–2.135)	<0.001	1.439 (1.079–1.904)	<0.05
Chest	1.382 (1.002–1.873)	<0.05	1.157 (0.833–1.581)	0.372
Stomach	1.276 (0.960–1.679)	0.087	1.105 (0.827–1.463)	0.493
Back	1.529 (1.165–1.993)	<0.05	1.330 (1.007–1.745)	<0.05
Waist	1.609 (1.231–2.122)	<0.001	1.518 (1.157–2.008)	<0.05
Buttocks	1.445 (1.049–1.960)	<0.05	1.244 (0.899–1.695)	0.176
Leg	1.500 (1.162–1.937)	<0.05	1.316 (1.015–1.709)	<0.05
Knees	1.536 (1.188–1.991)	<0.05	1.419 (1.094–1.844)	<0.05
Ankle	1.334 (0.992–1.772)	0.051	1.142 (0.845–1.527)	0.378
Toes	1.137 (0.800–1.580)	0.458	0.976 (0.683–1.364)	0.891
Neck	1.510 (1.146–1.974)	<0.05	1.353 (1.023–1.778)	<0.05

OR, odds ratio; 95% CI, 95% confidence interval.

*Model 1 adjusted for age and gender.

**Model 2 further adjusted for residence, marital status, smoking status, drinking status in the past year, health insurance, physical activity, self-health report, chronic disease, education level, and monthly household expenditure level.

**Table 4:** Association between vision impairment with co-occurrence of pain in multiple body parts stratified by age, gender and residence.

Number of pain occurrences in different body parts	OR (95% CI)	*p*-value	OR (95% CI)	*p*-value
Age, y^*^	≥45, <60		≥60	
Two	0.538 (0.349–0.796)	<0.05	0.538 (0.349–0.796)	<0.05
Three	0.927 (0.609–1.359)	0.712	0.927 (0.609–1.359)	0.712
Four	1.210 (0.781–1.797)	0.369	1.210 (0.781–1.797)	0.369
Five or more	1.578 (1.214–2.051)	<0.05	1.578 (1.214–2.051)	<0.05
Gender^**^	Male		Female	
Two	0.604 (0.311–1.070)	0.106	0.488 (0.267–0.822)	<0.05
Three	1.220 (0.670–2.080)	0.485	0.675 (0.361–1.158)	0.183
Four	1.790 (0.977–3.070)	<0.05	0.781 (0.394–1.397)	0.44
Five or more	1.620 (1.070–2.440)	<0.05	1.782 (1.278–2.495)	<0.05
Residence^***^	City/Town		Village	
Two	0.463 (0.177–1.000)	0.077	0.567 (0.344–0.885)	<0.05
Three	0.749 (0.285–1.630)	0.509	0.938 (0.580–1.440)	0.782
Four	0.996 (0.342–2.300)	0.993	1.220 (0.742–1.900)	0.407
Five or more	1.860 (1.090–3.150)	<0.05	1.620 (1.200–2.180)	<0.05

OR, odds ratio; 95% CI, 95% confidence interval.

*Adjusted for gender, residence, marital status, smoking status, drinking status in the past year, health insurance, chronic disease, education level and monthly Household Expenditure.

**Adjusted for age, residence, marital status, smoking status, drinking status in the past year, health insurance, chronic disease, education level and monthly Household Expenditure.

***Adjusted for age, gender, marital status, smoking status, drinking status in the past year, health insurance, chronic disease, education level and monthly Household Expenditure.

## 4 Discussion

Using nationally representative data of middle-aged and older adults in China, this study shows that individuals with VI have a higher prevalence of multi-site pain and coexistence of multi-site pain than those without VI. After adjusting for age, gender, place of residence, marital status, smoking status, drinking status, health insurance status, physical activity level, self-reported health status, chronic disease status, education level and household monthly expenditure, we found that VI was independently associated with the coexistence of two painful body sites (inversely) and with five or more painful body sites (positively), as well as with pain in eight different body sites (positively). And the association between VI and the coexistence of five or more painful body sites was not influenced by age, gender, or place of residence. To our knowledge, this is the first study to explore the relationship between VI and multi-site pain and coexistence of multi-site pain in LMICs.

In our study, we found a significant association between age and VI (*p <* 0.001), as shown in Table 1. However, the stratified analysis by age group (Table 4) revealed identical results across the two age groups (45–60 years and ≥ 60 years). This finding is somewhat unusual and requires careful consideration. First, we carefully checked the data quality and model settings. We confirmed that the data were accurately assigned to the correct age groups and that the model variables were consistently defined across all strata. The sample sizes in both age groups were sufficient (45-60 years: 4481; ≥ 60 years: 5759) and the results were stable. Several factors may contribute to this phenomenon. First, even though age is significantly associated with VI, the mechanisms linking VI to multi-site pain may be consistent across different age groups. Additionally, during the CHARLS survey process, participants in each age group and their family members (e.g., spouses) may share highly similar characteristics in other confounding variables, such as place of residence and socioeconomic status. This similarity may contribute to the consistent results observed across age groups. This similarity could lead to consistent results across age groups.

The underlying biological mechanisms linking VI and pain are highly complex, involving neuroinflammation, neuroimmune responses, and neural signal transduction. For instance, patients with neuromyelitis optica spectrum disorders (NMOSD) harbor aquaporin-4 (AQP4) antibodies, which can trigger immune-mediated neuroinflammatory responses, causing damage to the optic nerve and the spinal cord extensively (including demyelination of nerve fibers and axonal injury), leading to VI and sensory abnormalities in extensive body regions (including pain) (9–11). Additionally, during neuroinflammatory responses, microglia and astrocytes release pro-inflammatory cytokines (TNF- α /IL-1 β ) and chemokines (CCL2/CXCL1), which can modulate neuron–glia interactions to trigger the generation and transmission of pain signals (12). Other studies have shown that during neuroinflammatory responses, sensory neurons upregulate the expression of CSF1, IL-6, and CCL2 after injury. These factors indirectly activate neurons via the CNTF-STAT3-IL-6 axis, triggering pain perception and promoting the development of chronic pain (13). The aforementioned mechanisms may be linked to the association between VI and the coexistence of five or more painful body sites observed in our study.

Certain ocular diseases are often associated with pain in other body regions. For example, corneal diseases and ocular herpes zoster infections can cause facial pain due to the abnormal activation of the trigeminal nerve (14, 15). Anterior uveitis associated with autoimmune conditions is also commonly associated with waist pain (16). Studies have shown that 17–45% of patients with anterior uveitis also have HLA-B27 seropositive ankylosing spondylitis (AS), for which waist back pain is the most common symptom (17, 18). AS was not specifically mentioned in the 2020 CHARLS chronic disease data; thus, it is possible that the patients with the highest association of waist pain and VI in our study may belong to this group. In addition, other special types of uveitis, such as Behçet’s disease, also include joint pain as a common clinical symptom, which also supports the strong correlation between joint pain (such as in the fingers and knees) and VI observed in our study (19).

We considered several reasons for the lower likelihood of two coexisting pain sites in respondents with VI. First, VI may alter how the brain processes pain signals, changing an individual’s pain perception and responses. Research indicates that visual system damage can widely impact the brain’s neural networks, including areas related to pain processing, like the thalamus and cingulate gyrus (20–22). In some neuropathic pain conditions, abnormal neural signal conduction is linked to how patients perceive pain (23). VI might modify neural conduction pathways or regulate neurotransmitter release (24), thereby influencing pain signal integration and processing. This could make VI patients less sensitive to two pain sites compared to a single site, lowering the likelihood of two coexisting pain sites with VI. Second, people with VI may develop different pain cognition and coping mechanisms than those with normal vision due to long-term visual restrictions. They may focus more on their vision problems and either ignore or have a higher tolerance for pain in other body parts. Some psychology research shows that individual pain cognition and emotional responses significantly affect pain experience and reporting (25, 26). People with VI may use psychological suggestion and attention diversion to relieve or ignore mild to moderate pain. When the pain in two body sites is relatively mild, they are more likely to only report the more obvious one, thus reducing the reported rate of two coexisting pain sites. Lastly, VI limits patients’ daily activities and lifestyle, reducing the risk of multi-site pain caused by overactivity or poor posture. For instance, people with VI may avoid activities that can cause body collisions, twists, or overextensions. This protects certain muscles and joints, lessening pain from injuries or overuse (27). These behavior changes may indirectly lower the probability of multiple concurrent pain sites, particularly for two coexisting pain sites. However, this pain reduction does not affect the positive correlation between VI and multi-site (five or more sites) pain linked to neuroimmune diseases discussed earlier.

In summary, based on the findings of this study, we recommend early screening for systemic diseases related to autoimmune conditions in individuals with VI and coexisting multi-site pain. For patients with NMOSD and autoimmune-associated uveitis, the timely use of immunosuppressants and biologics can achieve comorbidity management, improve patient outcomes, and enhance quality of life. Furthermore, our findings underscore the public health significance of this issue, particularly in LMICs, where the coexistence of VI and multi-site pain poses a substantial health burden. These results offer valuable insights for policymakers and clinicians, aiding in the development of more effective health policies, the optimization of healthcare resource allocation, and the improvement of clinical practices. This not only enhances healthcare efficiency and service planning but also provides a foundation for future research and interventions aimed at addressing this critical public health challenge.

### 4.1 Limitations

This study also has certain limitations. First, as a cross-sectional study, it could not establish a causal relationship between VI and coexisting multi-site pain, which requires longitudinal studies to clarify. In future research, we will focus on conducting longitudinal studies to explore the causal relationship between VI and multi-site pain, as well as using more objective measurement methods to validate the findings of this study. Second, the CHARLS visual function assessment was conducted through interviewer-administered questionnaires, not objective measurements. Although previous studies have shown a good correlation between visual questionnaires and overall visual function, some differences may still exist (28). These biases could stem from individual differences in how interviewers interpret and report respondents’ visual status, potentially resulting in VI misclassification. Furthermore, the lack of objective measures, such as visual acuity tests, may limit the precision of VI assessment in this study. Future research could enhance the objectivity of VI measurement by incorporating standardized visual acuity tests or other objective evaluation methods. Similarly, the assessment of pain locations in CHARLS is based on respondents’ self-reports, which are susceptible to recall bias and subjective variations, such as differences in participants’ cultural backgrounds and understandings of pain definitions. Future studies would benefit from incorporating clinical validation of pain reports and employing standardized assessment methods. This would reduce biases and enhance the objectivity and reliability of pain measurement in relation to VI and other health outcomes. Third, although we adjusted for potential confounding factors such as age, gender, place of residence, self-reported health status, and chronic disease status, other residual confounding factors may still be present in our analysis. These confounders could stem from uncontrollable variables during data collection, such as participants’ psychological states and environmental factors. Fourth, while we hypothesize that neuroinflammatory mechanisms may underlie the associations between VI and specific pain sites (e.g., waist, fingers, knees), our cross-sectional design and lack of biomarker and neuroimaging data prevent us from conducting causal mediation analyses. Future longitudinal studies incorporating optical coherence tomography (OCT), cerebrospinal fluid cytokine profiling, and functional MRI may clarify these pathways. Finally, it should be noted that our study does not include some important chronic conditions like NMOSD or AS, which may be related to both VI and multi-site pain. The absence of these conditions in our analysis may potentially confound the findings. Future research should consider incorporating a broader range of chronic conditions to further explore and validate the associations identified in this study.

## 5 Conclusion

In this study, we found that among middle-aged and older adults in China, individuals with VI had a higher probability of pain across 15 body sites compared to those without VI. After adjusting for sociodemographic and health-related confounders, VI was inversely associated with the coexistence of two painful body sites but positively associated with five or more painful body sites. The association between VI with the coexistence of five or more painful body sites was not influenced by age, gender, or place of residence. These findings suggest that in LMICs, VI often coexists with multi-site pain, and patients with pain may benefit from ophthalmic care and comprehensive vision rehabilitation, thereby conserving medical resources. This has significant implications for improving the efficiency of healthcare systems, healthcare service planning, and the level of clinical practice.

## Data Availability

The original contributions presented in the study are included in the article/Supplementary material, further inquiries can be directed to the corresponding author.
